# Validity, reliability, and longitudinal measurement properties of the Asthma Daytime and Nighttime Symptom Diaries in patients with moderate-to-severe asthma

**DOI:** 10.1186/s41687-026-01066-5

**Published:** 2026-05-20

**Authors:** Tom Keeley, Dara O’Neill, Vita Dikariyanto, Gerasimos Dumi, Piper Fromy, Fedor Markov, Anna Richards, Rafael Alfonso-Cristancho

**Affiliations:** 1 Digital Measures, RWDMA, Development Sciences, GSK, 79 New Oxford Street, London, WC1A 1DG UK; 2 Patient-Centered Solutions, IQVIA, Barcelona, Spain; 3 Patient-Centered Solutions, IQVIA, London, UK; 4 Patient-Centered Solutions, IQVIA, Athens, Greece; 5 SeeingTheta, Saumur, France; 6 Global Safety, GSK, London, UK; 7 Global Real-World Evidence and Health Outcomes Research, GSK, London, UK; 8 Global Real-World Evidence and Health Outcomes Research, GSK, Collegeville, PA USA; 9 Patient Centered Outcomes, Global Real-World Evidence and Health Outcomes Research, GSK, 79 New Oxford Street, London, WC1A 1DG UK

**Keywords:** Patient-reported outcome, Moderate asthma, Severe asthma, Psychometrics, Meaningful change, Real-world evidence, Randomized controlled trial, Symptom diary

## Abstract

**Background:**

The Asthma Daytime and Nighttime Symptom Diaries (ADSD and ANSD) were developed to provide an accurate and standardized instrument for assessing patient-reported severity of asthma symptoms. While previous evidence supports the content validity and cross-sectional measurement properties of the ADSD and ANSD, the US Food and Drug Administration (FDA) qualification statement for these tools recommended further evaluation of their longitudinal measurement properties and the interpretation of within-patient meaningful change (WPMC). Therefore, two complementary studies were designed to assess cross-sectional and longitudinal measurement properties and estimate thresholds for WPMC for the ADSD/ANSD in patients with moderate-to-severe asthma, responding to measurement gaps identified in the FDA statement.

**Methodology:**

Quantitative evaluation of ADSD/ANSD (score range 0 to 10) measurement properties was performed in a real-world observational study (RWS; ≥16 years old; moderate-to-severe asthma) and a randomized controlled trial (RCT; ≥12 years old; severe asthma with type 2 inflammation). Cross-sectional and longitudinal measurement properties were assessed and WPMC thresholds estimated.

**Results:**

A total of 576 patients with moderate-to-severe asthma were recruited across both studies. The RWS (*n* = 241) and RCT (*n* = 335) both found the ADSD/ANSD to have high internal consistency and test-retest reliability with Cronbach’s α-coefficients ≥0.94 and intraclass correlation coefficients ≥0.90. Evidence in support of the unidimensional scoring solution for both the ADSD and ANSD was established via confirmatory factor analysis across timepoints and studies, and evidence of construct validity was demonstrated via convergent and known-groups validity testing. In the blinded RCT analysis, the threshold for WPMC was estimated at 1.2 for the ADSD and 1.5 for the ANSD. In the RWS, the WPMC threshold for within-patient improvement was estimated to be from 0.6 to 1.3 for ADSD and from 0.8 to 1.4 for ANSD across 6- and 10-week intervals.

**Conclusions:**

This study directly addresses the evidence gaps identified by the FDA, demonstrating that the ADSD and ANSD are reliable and valid tools for the measurement of daily symptoms in patients with moderate-to-severe asthma, and are capable of detecting WPMC. Values derived from the RCT analysis support a recommendation for thresholds for meaningful within-patient change of 1.2 and 1.5 for the ADSD and ANSD, respectively.

**Clinical trial registration:**

GSK ID: 217640; NCT04719832.

**Supplementary Information:**

The online version contains supplementary material available at 10.1186/s41687-026-01066-5.

## Background

Patient-reported outcome (PRO) measures can provide valuable insights into asthma control, alongside outcomes such as forced expiratory volume in 1 s, peak flow, and exacerbation rates [1–3]. Data from the 2011–2013 US National Health and Wellness Survey showed that over half of patients with asthma in the USA report poor symptom control despite treatment [4]. Reductions in the frequency and severity of symptoms are a key indicator of improved asthma control. Many of these symptoms can only be assessed by patients themselves using PRO measures [5]. A range of PRO tools, such as the Asthma Control Questionnaire (ACQ-5/ACQ-6), the Asthma Quality of Life Questionnaire, and the St George’s Respiratory Questionnaire (SGRQ) have been validated for use in asthma clinical trials and in clinical settings; however, available tools largely assess asthma control and quality of life more broadly, rather than symptom severity specifically [5]. Existing measures are not designed to capture the nuances of the known daily symptom variation in asthma [6], and no instrument focuses solely on symptoms at night. Each of these are crucial for understanding symptom severity over time, and are better suited to group comparisons in a research setting; as a result, PRO measures are not routinely used in clinical practice [5].

The Asthma Daytime Symptom Diary (ADSD) and Asthma Nighttime Symptom Diary (ANSD) were originally developed by the PRO Consortium’s Asthma Working Group at the Critical Path Institute to address the need for a standardized daily PRO assessment of the severity of the core symptoms of asthma. Both PRO measures comprise six items evaluating the severity of a set of core asthma symptoms, including daytime (ADSD) and nighttime (ANSD) incidence of difficulty breathing, wheezing, shortness of breath, chest tightness, chest pain, and cough. US-based patients with mild-to-severe asthma, aged 12 years and above, participated in qualitative (*n* = 120) [7] and quantitative (*n* = 219) [8] studies to inform initial development, establish content validity, and evaluate cross-sectional measurement properties of the ADSD and ANSD. To ensure reliable, valid and accurate outcome measurement, the ADSD and ANSD were developed in line with current recommendations from published literature for PRO measurement amongst patient populations with asthma as well as US Food and Drug Administration (FDA) guidelines [8].

The FDA qualification statement for the ADSD and ANSD specifically supported their use in drug development and regulatory review but noted the need for further evaluation of the longitudinal measurement properties (ability to detect change) and the interpretation of within-patient meaningful change (WPMC) before the qualification for the ADSD and ANSD could be expanded to include use as primary or secondary endpoint measures in confirmatory studies [9]. This article reports the quantitative assessment of the cross-sectional and longitudinal psychometric properties and estimates of WPMC for the ADSD and ANSD in patients with moderate-to-severe asthma, conducted in response to this guidance from the FDA.

## Methods

This article reports results from two studies: (1) a real-world study (RWS) — a prospective, noninterventional online survey in adults with moderate-to-severe asthma (self-reported asthma of ≥ 2 years duration with asthma medication(s) prescription verification step included during recruitment) in an observational setting in the US, UK, and Germany (GSK ID: 214135), and (2) a randomized controlled trial (RCT) — an in-trial psychometric evaluation in a population of adults and adolescents with severe asthma with type 2 inflammation (i.e. at risk of exacerbations, defined as ≥ 2 exacerbations in the previous 12 months and with peripheral blood eosinophil count ≥ 300 cells/µL in the last 12 months or ≥ 150 cells/µL at screening; GSK ID: 217640 [NCT04719832]). The full eligibility criteria applied to each study are detailed in Table E1.

The primary objective of these analyses was to assess cross-sectional and longitudinal psychometric properties of the ADSD and ANSD in real-world (GSK ID: 214135) and controlled conditions (GSK ID: 217640 [NCT04719832]). The intention of assessing the performance of the tools across two types of study was to offer complementary insights in different settings and establish confidence in the robustness of the evidence by replicating findings across settings.

### Study procedures

#### RWS (214135)

**Fig. 1:**
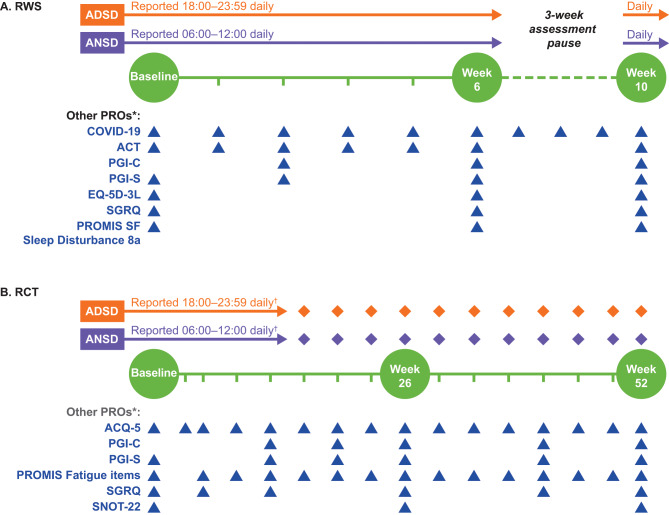
Study design overviews of the RWS (**A**) and RCT (**B**). *Completed on Days 1 and 7 during Baseline and Day 7 in all other indicated weeks (except EQ-5D-3L and SGRQ, which were completed once per week, on any day, in all indicated weeks); ^†^the ADSD and ANSD were completed daily during the run-in phase through to Week 16 of the RCT, then were completed for 1 full week during the week preceding each visit thereafter. ACQ-5, Asthma Control Questionnaire 5-item; ACT, Asthma Control Test; ADSD, Asthma Daytime Symptom Diary; ANSD, Asthma Nighttime Symptom Diary; EQ-5D-3L, EuroQoL 5-Dimensions 3-Levels; PGI-C, Patient Global Impression of Change (Day & Night measures in RWS only); PGI-S, Patient Global Impression of Severity (Day & Night measures in RWS only); PROMIS SF, Patient-Reported Outcomes Measurement Information System Short Form; RCT, randomized controlled trial; RWS, real-world study; SGRQ, St George’s Respiratory Questionnaire; SNOT-22, Sino-Nasal Outcome Test-22

The RWS was a prospective online survey assessing the psychometric performance of the ADSD and ANSD in patients with moderate-to-severe asthma recruited through patient panels, support groups, and patient forums in the USA, Germany, and UK. The ADSD and ANSD questionnaires were administered daily for 6 weeks, followed by a 3-week break before a final week of assessment. This break was included to help attain understanding of whether patient engagement and patient completion differed when assessment occurred habitually for a period and when re-initiated after a break; this understanding may also inform future study approaches, as it can aid assessment of whether these measures are suitable for use in decentralized clinical trials. Eligible patients had to download the electronic clinical outcome assessment (eCOA) app, complete the required training, and provide at least one datapoint on the ADSD or ANSD to be included in the analysis sample. The eCOA comprised 11 different measures, as summarized in Fig. 1A. Analyses were performed on data collected at Baseline, Week 6, and Week 10, with test-retest reliability analyses also using data from Week 3.

#### RCT (217640)

The RCT included assessment of the psychometric performance of the ADSD and ANSD in patients enrolled in SWIFT-1 (NCT04719832), a 52-week, randomized, double-blind, placebo-controlled, parallel-group, multicenter study of depemokimab 100 mg subcutaneous every 6 months in patients with severe asthma with type 2 inflammation (i.e. a history of ≥ 2 exacerbations in the previous 12 months and a peripheral blood eosinophil count ≥ 300 cells/µL in the last 12 months or ≥ 150 cells/µL at screening). As part of the trial, patients completed a range of electronically-administered PRO measures assessing disease severity and impact, and analyses were performed on data collected at Baseline, Week 26, and Week 52, with test-retest reliability analyses also using data from Weeks 12, 16, and 20. Several PROs were used as anchors (Fig. 1B). These psychometric analyses of the ADSD and ANSD were blinded to treatment.

### Data source and collection

#### ADSD and ANSD

The ADSD and ANSD measure the severity of six asthma symptoms including difficulty breathing, wheezing, shortness of breath, chest tightness, chest pain, and cough. Each item is rated by respondents on an 11-point numeric rating scale ranging between 0 (“None”) and 10 (“As bad as you can imagine”). Respondents completed the ADSD in the evening before going to bed to assess their asthma symptoms during that day and completed the ANSD in the morning upon waking to assess their asthma symptoms during the prior night. The ANSD collection window was between 6:00 am and 11:59 am and the ADSD collection window was between 6:00 pm and 11:59 pm. Scoring for the ADSD and ANSD focused on the derivation of daily and weekly *total scores*. *Daily scores* were calculated by averaging responses across all six items for Days 1 to 7 of each study week; and *weekly summary scores* were calculated each study week using the daily scores from within that period. For a valid weekly summary score to be calculated, patients were required to provide at least 50% of daily scores in that week, i.e., at least 4 out of 7 daily scores. Missing data were not imputed; this rule was applied separately for the ADSD and ANSD. In the RWS, the assessment windows were defined as Baseline (Day 1 to 7 at Baseline), Week 3 (Day 15 to 21), Week 6 (Day 36 to 42), and Week 10 (Day 64 to 70). In the RCT, the ADSD and ANSD assessment windows were defined as the weekly period immediately preceding the clinic visit.

#### Additional PRO measures

PRO assessment schedules for each study are detailed in Fig. 1A (RWS) and Fig. 1B (RCT), and an overview of each PRO can be found in Table E2. PROs included by study were: ACQ-5 (RCT only), Asthma Control Test (ACT; RWS only), EuroQoL 5-Dimensions 3-Levels (EQ-5D-3 L, RWS only), Patient Global Impression of Severity (PGI-S; RWS and RCT), Patient Global Impression of Change (PGI-C; RWS and RCT), Patient-Reported Outcome Measurement Information System Short Form (PROMIS SF; RWS only), Sleep Disturbance 8a (RWS only), PROMIS Fatigue items assessing the concept of ‘Energy’ (RCT only), SGRQ (RWS and RCT), Sino-Nasal Outcome Test-22 (SNOT-22; RCT only), and a PRO questionnaire on COVID-19 symptoms and test results (RWS only) were completed. In the RWS, daytime and nighttime versions of the PGI-S (including PGI-S Day and PGI-S Night) and PGI-C (including PGI-C Day and PGI-C Night) were used.

### Data analyses and assessment measures

#### Structural validity

A set of confirmatory factor analyses (CFAs) was carried out to evaluate the unidimensional latent six-item structure of the ADSD and ANSD at different timepoints, as had been previously established by Gater et al. [8]. Separate CFAs to examine unidimensional models (all items set to load on a single factor) were carried out for each of the ADSD and ANSD on the target day of each key assessment period (RWS: Day 7 of Baseline, Week 6, and Week 10; RCT: Day 7 of Baseline, Week 26, and Week 52), and a (one-factor) model was tested for ADSD and ANSD separately at each timepoint. Fit indices were calculated to assess model goodness-of-fit, specifically chi-square, the standardized root mean residual (SRMR) as a measure of absolute fit, the root mean square error of approximation (RMSEA) as a parsimony-adjusted alternative to the SRMR, and the comparative fit index (CFI). Acceptable fit values were close to 0.08 or under for SRMR, close to 0.06 or under for RMSEA, and close to 0.95 or higher for CFI [10].

#### Internal consistency

Internal consistency of the ADSD and ANSD measures were evaluated in the RWS and RCT to assess the coherence of the items to form a reliable measurement scale. Cronbach´s alpha (α) and α-if-item-deleted were estimated to evaluate internal consistency; McDonald’s omega (ω) values were additionally estimated in the RCT.

#### Test-retest reliability

Score reproducibility across select timepoints was examined in the RWS and RCT. For the RWS, test-retest reliability was assessed between Baseline–Week 3, Weeks 3–6, and Weeks 6–10. For the RCT, the periods were Weeks 12–20, Weeks 16–20, and Weeks 20–26. These timepoints reflected the shortest intervals at which the PGI-S and ADSD and ANSD were concurrently collected. In the case of the RCT, the selection of these timepoints also took account of clinical guidance that earlier timepoints in the trial would be more likely to have been a period of change in the patient symptom severity, hindering the derivation of a stable patient subgroup as befits an analysis of test-retest reliability. Two-way random, absolute agreement, single measure intraclass correlation coefficients (ICCs) [11] were calculated for both the ADSD and ANSD weekly summary scores. ICC values of 0.50 to 0.90 were considered to represent moderate to good reliability and values > 0.90 to represent excellent reliability [12]. These analyses were performed on patient subcohorts with stable asthma severity, determined via their responses to comparator/anchor measures. In both studies, stable condition was defined using PGI-S. For the RWS, three sets of analysis were performed, focusing on change between Baseline (Day 7 with 1-week recall period) and Week 3, between Weeks 3 and 6, and between Weeks 6 and 10. For each analysis, only those who reported the same PGI-S severity level across the assessment interval were included. For the evaluation of stability of the ADSD and ANSD scores in the RCT, analyses focused on patients who were identified as being stable based on their responses to the PGI-S across Week 12–20 and Week 20–26. Given the length of the assessment intervals across which PGI-S could be utilized in assessing test-retest reliability in the RCT, a supplementary approach utilized the ACQ-5 item 2 (“On average, during the past week, how bad were your asthma symptoms when you woke up in the morning?”) as a basis for identifying stable patients during the Week 16 to Week 20 interval. Only those patients reporting “no change” on this item from Week 16 to 20 were included.

#### Differential item functioning (DIF)

DIF was assessed in the RWS to determine whether patients from different groups but with similar underlying symptom severity had a different probability of giving a particular response on the ADSD and ANSD. The following group comparisons were undertaken: confirmed/suspected COVID-19 infection during course of study versus no COVID-19 infection reported (with noninfected as the reference group); Global Initiative for Asthma (GINA) step 4 versus biologic/nonbiologic GINA step 5 (with step 5 as the reference group) on Day 7 of Baseline; and UK- versus USA-based patients (with USA as the reference category) on Day 7 of Baseline. Given that the sample size for Germany was below 50, DIF comparisons involving this subsample are not reported. Correction for multiple testing was applied per comparison to reduce the risk of Type I error inflation using the Benjamini–Hochberg method [13]. 

#### Convergent and divergent validity

Correlation with other PRO measures was used to evaluate convergent and divergent validity for the ADSD and ANSD. At Baseline, Week 6, and Week 10 of the RWS, correlations were examined between ADSD and ANSD weekly summary scores and ACT, SGRQ, EQ-5D-3L, and PROMIS SF Sleep Disturbance 8 A. At Baseline, Week 26, and Week 52 of the RCT, correlations were examined between ADSD and ANSD weekly summary scores and SGRQ (activities, symptom, and item 7 “how many good days have you had” scores), ACQ-5 (total score), SNOT-22 (total, function, and sleep scores), and PROMIS Fatigue item 1 (“how often did you run out of energy?”) score. For comparisons using total/domain scores, correlations were estimated; choice of correlation coefficient was based on evaluation of skewness, with Spearman rank coefficients used in lieu of Pearson where skewness was severe. For correlations using single items as comparators, polyserial correlations were estimated. It was expected that there would be moderate to high correlations between assessments of similar constructs (i.e., ≥ 0.30) [14]. While no truly ‘divergent’ measures were administered, some measures were expected to be less related to the constructs measured by the ADSD (e.g., EQ-5D Visual Analog Scale and EQ-5D-3L Usual Activities; SGRQ Activity and Impact scores) and ANSD (e.g., EQ-5D-3L Anxiety/Depression, Self-Care and Usual Activities).

Known-groups validity was assessed to understand the degree to which a measure can distinguish between groups of patients hypothesized to be different in the concept of interest (e.g., patients with no symptoms [assessed via PGI-S] vs. patients with mild symptoms [also assessed via PGI-S]).

In the RWS, comparisons to the ADSD and ANSD were completed cross-sectionally for PGI-S, SGRQ, and ACT groupings at Baseline, Week 6, and Week 10 using collapsed categories. Additional details of known-groups validity assessment are described in the Supplementary methods.

Within each definition, between-groups effect sizes were estimated for each consecutive category pairing, with the effect size values calculated as the mean difference in ADSD and ANSD weekly summary score between groups divided by the pooled standard deviation (SD) of those groups. Monotonicity in the group differences (e.g., did the groups show expected trends in the difference across the severity levels assessed by the ADSD and ANSD) was a key basis of interpretation of this analysis. Significance tests (Kruskal–Wallis H test with alpha = 0.05 level) were additionally performed with the known-groups as the independent variable and ADSD and ANSD scores as dependent variables.

#### Sensitivity to change

Sensitivity to change was examined in both studies to assess the ability of the ADSD and ANSD to accurately reflect where change was understood to have occurred (as determined via patients’ PGI-S/PGI-C scores).

This was assessed using analysis of covariance (ANCOVA), with the dependent variable as the change from Baseline in ADSD and ANSD weekly summary score data. The models included the “responder” factor as a fixed factor and the analyses controlled for the PRO Baseline scores. Patients with missing data on the ADSD/ANSD change score or responder factor were excluded, and the ANCOVA assumptions included independence of observations, linear covariate effects with homogeneous slopes across anchor groups, and homoscedastic, approximately normal residuals. While significant p-values were considered supportive of expected differences in mean change scores, within- and between-group effect sizes were prioritized when evaluating patterns of change.

A combination of analytic (using ANCOVA) and visualization approaches (waterfall plots) were used. Anchor instruments were utilized to define responder groups using change from Baseline in ADSD and ANSD weekly summary scores at Week 6 and Week 10 for RWS data and Week 26 and Week 52 for RCT data as the dependent variable, with the PGI-S (as a prospectively measured item) serving as the primary anchoring method and the PGI-C (as a retrospectively measured item) serving as a supportive approach. Significance values were considered supportive of monotonic differences expected in mean change scores and effect sizes of change. Both within- and between-groups effect sizes were assessed.

#### WPMC

Assessment of WPMC was also conducted in both studies to determine the amount of individual level change over a predefined period that can be deemed a meaningful benefit. Thresholds for WPMC were estimated using anchor-based approaches, supplemented with visualization strategies (empirical cumulative distribution function and probability density function curves) and distribution-based approaches (half Baseline SD and standard error of measurement). WPMC thresholds were estimated for change in both ADSD and ANSD weekly summary scores. In the RWS, PGI-S Day, PGI-S Night, PGI-C Day, and PGI-C Night were employed as anchors for defining the direction and magnitude of patient change between Baseline and Weeks 6 and 10. Analyses were performed using both uncollapsed and collapsed anchor change score categorizations. In the RCT, both uncollapsed and collapsed categorizations of change scores on the PGI-S and PGI-C between Baseline and Weeks 26 and 52 were used as anchors to evaluate meaningful change. The uncollapsed versions were prioritized in triangulation of the different estimates as they may allow detection of more minimal levels of meaningful change, but the collapsed anchor versions served independent utility where they helped achieve clearer differentiation between patients who had and had not experienced meaningful change.

For both studies, results of the anchor-based analyses were considered primary when making decisions regarding the WPMC threshold. Evaluation based on the PGI-S, as a prospectively measured item, was given primacy as an anchor over the PGI-C (due to the latter being a retrospectively measured item), with collapsed categories including ‘Improved’, ‘No change’ and ‘Worsened’. If the correlation coefficient was greater than 0.3, the anchor was considered adequate [15]. Analyses with anchors whose correlations with the ADSD and ANSD were < 0.3 were still performed but not considered in the estimation of the responder definitions. Anchor-based evaluations were also informed by evaluation of distribution-based and data visualizations. Findings from the RCT were the main assessment for WPMC, given the known changes occurring in this study; as the RWS was noninterventional, findings established from it were considered supportive.

#### Ethics

Institutional review board approval for the RWS was obtained from Western Copernicus Group Independent Review Board (tracking number 20210121). Ethical approval for the RCT was obtained as part of the SWIFT-1 trial (NCT04719832); local ethical approval was obtained at each study site.

## Results

### Patient population

The RWS and RCT (depemokimab-treated and placebo-treated) included 241 and 335 patients, respectively. The RWS included patients with moderate-to-severe asthma, while the RCT included patients with severe asthma with type 2 inflammation. All patients were from North America and/or Europe. Demographic characteristics of the study populations are comparable (Table 1).

**Table 1:** Sociodemographic and clinical characteristics at Baseline

Sociodemographic and clinical characteristics	RWS (214135) *N* = 241	RCT (217640) *N* = 335
Age, years		
Mean (SD) Min–max	41.5 (11.47) 16–77	54.0 (14.62) 14–79
Sex, n (%)	*N* = 236	
Male Female	52 (22.0) 184 (78.0)	140 (41.8) 195 (58.2)
ACT classification, n (%)	*N* = 191	NA
Very poorly controlled (≤ 15) Poorly controlled (> 15 ≤ 19) Well controlled (≥ 20)	- 141 (73.8)* 50 (26.2)*	
GINA step, n (%) 3 4 5	*N* = 234 113 (48.3) 50 (21.4) 71 (30.3)^†^	NA
Smoking status,^‡^ n (%)	*N* = 236	
Nonsmoker Former Smoker	199 (84.3) 33 (14.0) 4 (1.7)	250 (74.6)^§^ - 85 (25.4)^§^

*The RWS categorized ACT score as poorly controlled (≤ 19) and well controlled (≥ 20) only;

^†^a biologic treatment was used in 23 (9.8%) of patients receiving GINA step 5 therapy in the RWS; ^‡^smokers were included if they smoked ≤ 5 cigarettes per day, former smokers were defined as patients who had stopped smoking at least 6 months before their first visit and had a smoking history of ≤ 10 pack years; ^§^the RCT categorized current and former smokers as smokers

ACT, Asthma Control Test; GINA, Global Initiative for Asthma; NA, not available; RCT, randomized controlled trial; RWS, real-world study; SD, standard deviation

### Structural validity

**Fig. 2:**
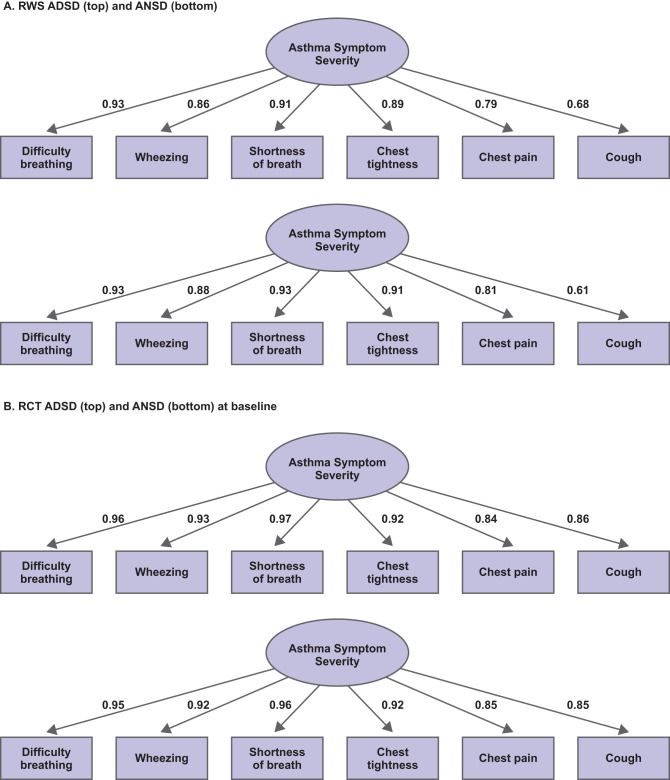
One-factor confirmatory factor analysis path diagrams for the RWS (**A**) and RCT (**B**). Factors (latent variables) are represented as ellipses and observed items (diary questions) as rectangles. ADSD, Asthma Daytime Symptom Diary; ANSD, Asthma Nighttime Symptom Diary; RCT, randomized controlled trial; RWS, real-world study

Evidence in support of the unidimensional scoring solution for both the ADSD and ANSD was established via CFA at Baseline and follow-up. Individual item loadings on this one-factor structure were similar across timepoints and across the two measures in the one-factor CFA models for both the RWS and RCT (loading range 0.61–0.98; Table E3 and Fig. 2). Goodness-of-fit indices in the RWS approached or exceeded recommended thresholds through the CFI (all ≥ 0.91) and SRMR (all ≤ 0.04), although significant chi-squares and RMSEA values ≥ 0.12 were observed at Baseline. However, there was evidence of improved fit at Weeks 6 and 10 (e.g., RMSEA = 0.09 for ADSD and 0.07 for ANSD each at Week 6 and 10). The RCT showed highly comparable goodness-of-fit results in evaluations at Baseline, Week 26, and Week 52.

### Reliability of the ADSD and ANSD

The ADSD and ANSD showed high internal consistency (Cronbach’s α- ≥0.94) in both the RWS and RCT studies (Table 2). Across studies and timepoints, α-if-item-deleted results showed that deleting any single item produced no material increase in α (maximum increase ≈ 0.01), and in several cases α decreased (maximum decrease ≈ 0.02), suggesting that no item was trivially redundant and that all items contributed to the total score (Table E4). McDonald’s ω was also analyzed. In the RCT, ω indicated good reliability for the ADSD total score (> 0.7 at all timepoints except Week 52; ω = 0.69). ANSD total scores showed good internal consistency at Baseline and Week 52 but were marginally below the 0.7 guideline at Week 26 (ω = 0.68). The ADSD and ANSD showed high test-retest reliability with ICCs of ≥ 0.90 in both the RWS and RCT studies across all time intervals examined.

**Table 2:** The ADSD and ANSD showed high internal consistency and good test-retest reliability

Internal consistency in the RWS: Cronbach’s α-coefficient (95% CI)				
ADSD			ANSD	
Baseline (*n* = 205)	0.94 (0.93, 0.95)		Baseline (*n* = 191)	0.95 (0.94, 0.96)
Week 6 (*n* = 174)	0.96 (0.95, 0.97)		Week 6 (*n* = 162)	0.96 (0.95, 0.97)
Week 10 (*n* = 163)	0.97 (0.96, 0.97)		Week 10 (*n* = 163)	0.97 (0.96, 0.97)

Internal consistency in the RCT: Cronbach’s α-coefficient (95% CI)				
ADSD		ANSD		
Baseline (*n* = 328)	0.95 (0.95, 0.96)	Baseline (*n* = 300)		0.95 (0.95, 0.96)
Week 26 (*n* = 189)	0.96 (0.95, 0.97)	Week 26 (*n* = 178)		0.95 (0.94, 0.96)
Week 52 (*n* = 101)	0.96 (0.94, 0.97)	Week 52 (*n* = 77)		0.95 (0.93, 0.96)

Test-retest reliability among patients with stable asthma (defined by PGI-S) in the RWS: ICC (95% CI)				
ADSD		ANSD		
Baseline – Week 3 (*n* = 90)	0.92 (0.87, 0.95)	Baseline – Week 3 (*n* = 80)		0.90 (0.85, 0.94)
Week 3–6 (*n* = 89)	0.93 (0.90, 0.96)	Week 3–6 (*n* = 72)		0.95 (0.92, 0.97)
Week 6–10 (*n* = 80)	0.96 (0.94, 0.97)	Week 6–10 (*n* = 63)		0.93 (0.89, 0.96)

Test-retest reliability among patients with stable asthma (defined by PGI-S) in the RCT: ICC (95% CI)				
ADSD		ANSD		
Week 12–20 (*n* = 154)	0.96 (0.94, 0.97)	Week 12–20 (*n* = 146)		0.96 (0.95, 0.97)
Week 20–26 (*n* = 115)	0.97 (0.95, 0.98)	Week 20–26 (*n* = 102)		0.98 (0.96, 0.98)

For the test-retest reliability analyses reported in this table, stable patients were defined as patients with an unchanged PGI-S response across the stated interval. Similar results were observed when stability was defined by alternative definitions (data not shown)

ADSD, Asthma Daytime Symptom Diary; ANSD, Asthma Nighttime Symptom Diary; CI, confidence interval; ICC, intraclass correlation coefficient; PGI-S: Patient Global Impression of Severity; RCT, randomized controlled trial; RWS, real-world study

### Convergent and divergent validity of the ADSD and ANSD

Correlations between ADSD and ANSD and the ACQ-5 (RCT only), ACT (RWS only), PROMIS SF Sleep Disturbance 8a (RWS only), PROMIS Fatigue item 1 (question: how often did you run out of energy?; RCT only), and SGRQ subscores were in line with a priori expectations and demonstrated acceptable convergent validity in both the RWS and RCT (Table 3). More moderate correlations were observed with the EQ-5D-3L (utility) in the RWS and SNOT-22 (rhinosinusitis) measures in the RCT.

**Table 3:** Convergent/divergent validity of ADSD and ANSD weekly summary scores with other PRO measures

RWS													
		ADSD						ANSD					
		Baseline		Week 6		Week 10		Baseline		Week 6		Week 10	
		*n*	*r*	*n*	*r*	*n*	*r*	*n*	*r*	*n*	*r*	*n*	*r*
Convergent validity	ACT total score	186	**-0.78**	147	**-0.77**	141	**-0.74**	173	**-0.78**	137	**-0.76**	141	**-0.72**
	SGRQ total score*	204	**0.67**	171	**0.61**	160	**0.65**						
	SGRQ symptom score*	204	**0.70**	171	**0.66**	160	**0.77**	191	**0.66**	160	**0.69**	160	**0.70**
	SGRQ ‘good days’ item^†^	204	**-0.60**	171	**-0.69**	160	**-0.68**						
	PROMIS SF Sleep Disturbance 8a total score*							163	*0.37*	135	**0.51**	136	**0.58**
Divergent validity	EQ-5D VAS*	204	-0.44	171	-0.42	160	-0.49						
	EQ-5D-3L Usual Activities^†^	200	*0.50*	169	*0.55*	160	0.49	187	0.43	158	*0.56*	160	0.46
	SGRQ Activities score*	204	*0.55*	171	0.44	160	0.45						
	SGRQ Impact score*	204	*0.63*	171	*0.61*	160	*0.65*						
	EQ-5D-3L Anxiety/Depression^†^							191	0.35	160	0.31	160	0.40
	EQ-5D-3L Self-Care^†^							188	*0.55*	160	0.45	160	*0.56*

**RCT**													
		**ADSD**						**ANSD**					
		**Baseline**		**Week 26**		**Week 52**		**Baseline**		**Week 26**		**Week 52**	
		**n**	**r**	**n**	**r**	**n**	r	**n**	**r**	**n**	**r**	**n**	**r**
Convergent validity	ACQ-5 total score*	325	**0.67**	179	**0.76**	90	**0.76**	298	**0.69**	159	**0.74**	67	**0.74**
	SGRQ symptom subscore*	325	**0.61**	177	**0.71**	90	**0.63**	298	**0.61**	158	**0.66**	67	**0.63**
	SGRQ item 7: How many good days have you had?^†^	325	*-0.45*	177	*-0.31*	90	*-0.26*						
Divergent validity	SGRQ activity score*	325	0.41	177	0.45	90	0.39						
	SNOT-22 total score*	323	0.45	177	*0.51*	90	0.48	296	0.44	158	*0.55*	67	*0.56*
	SNOT-22 function score*	323	0.38	177	0.47	90	0.39						
	SNOT-22 sleep score*							296	0.39	158	0.49	67	0.49
	PROMIS Fatigue item 1: How often did you run out of energy?^†^	189	0.47	88	*0.66*	37	*0.65*	164	0.49	79	*0.64*	26	*0.72*

*Spearman correlation; ^†^polychoric/polyserial correlation

**Bold text** shows correlations of ADSD and ANSD weekly summary scores with the convergent validity measures equal to or greater than 0.5; underlined text shows correlations of ADSD and ANSD weekly summary scores with the divergent validity measures lower than 0.5; *italicised text* shows correlations that did not meet the predefined thresholds. The difference in comparisons evaluated for the ADSD and ANSD was driven by the fact that the majority of suitable comparator scales included components focused on daily activities, which are much less applicable during nighttime hours

ACQ-5, Asthma Control Questionnaire-5; ACT, Asthma Control Test; ADSD, Asthma Daytime Symptom Diary; ANSD, Asthma Nighttime Symptom Diary; EQ-5D-3L, EuroQoL 5-Dimensions 3-Levels; n, number of patients; PRO, patient-reported outcome; PROMIS SF, Patient-Reported Outcomes Measurement Information System Short Form; r, correlation coefficient; RCT, randomized controlled trial; RWS, real-world study; SGRQ, St George’s Respiratory Questionnaire; SNOT-22, Sino-Nasal Outcome Test-22; VAS, Visual Analog Scale

### Known-groups validity of ADSD and ANSD

**Fig. 3:**
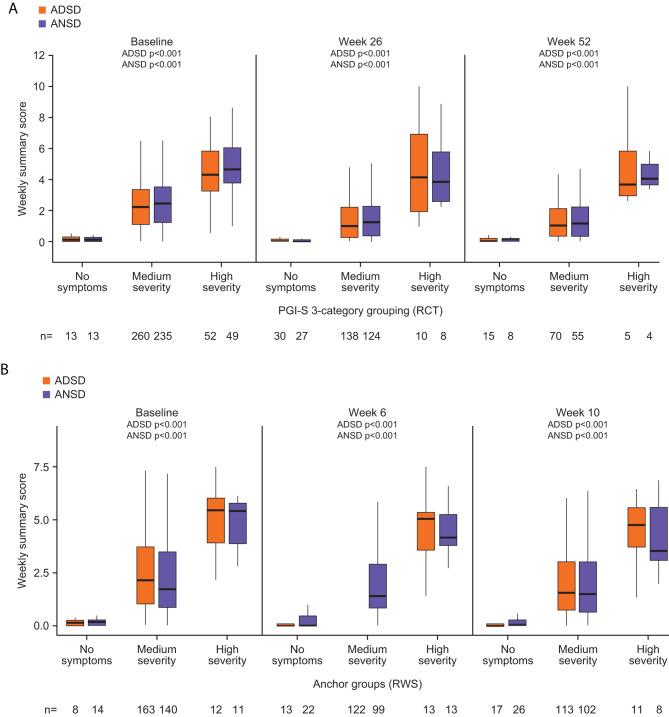
ADSD and ANSD demonstrate ability to differentiate between known-groups defined by PGI-S category in the RCT (**A**) and RWS (**B**). PGI-S groups defined as ‘No symptoms,’ ‘Medium severity’ (including mild and moderate), and ‘High severity’ (including severe and very severe) collapsed categories (3 categories). *P*-values denote the significance level of the Kruskal–Wallis H test among groups. A *p*-value < 0.05 indicates that the ADSD or ANSD scores differ significantly among the three groups at the given timepoint. ADSD, Asthma Daytime Symptom Diary; ANSD, Asthma Nighttime Symptom Diary; PGI-S, Patient Global Impression of Severity (Day & Night measures in RWS only); RCT, randomized controlled trial; RWS, real-world study

For the blinded RCT data, there was a pattern of monotonic increase in ADSD and ANSD summary scores across the PGI-S–defined groups (consistent with increasing severity in these groupings). The between-group effect sizes were all large for the PGI-S (absolute range: 0.97–2.13), and all group comparisons were statistically significant (*p* < 0.001) at Baseline, Week 26, and Week 52 (Fig. 3A). In the RWS, similar patterns were seen with groups defined by responses to the SGRQ item “How would you describe your chest condition” and to the PGI-S at Baseline, Week 6, and Week 10 (Fig. 3B).

### DIF

After correction for multiple testing, no statistically significant evidence of DIF was observed in RWS patients with versus without COVID-19, for those receiving GINA step 4 versus GINA step 5 therapy, or between UK- versus US-based patients. Additional details of the DIF results prior to multiple testing are described in the Supplementary results.

### Sensitivity to change

**Fig. 4:**
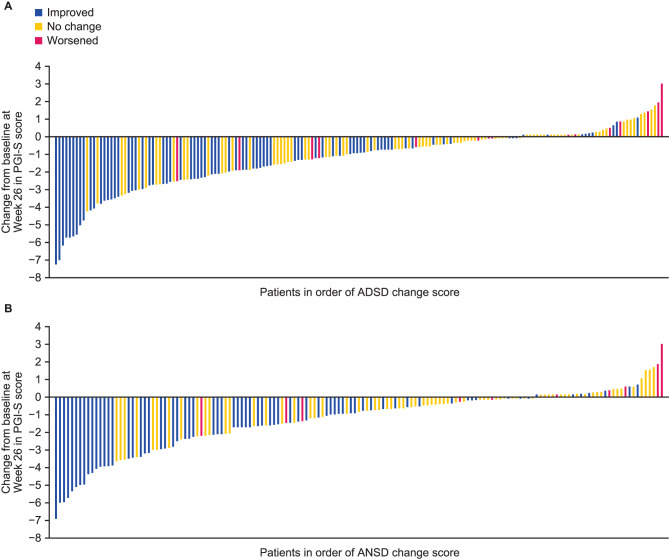
Most patients who experienced an improvement in PGI-S score during the RCT also showed improvements in ADSD (**A**) and ANSD (**B**) scores. ‘Improved’ is defined as patients who reported at least a 1-point improvement on the PGI-S between Baseline and Week 26 (ADSD *n* = 80; ANSD *n* = 70); ‘No Change’ is defined as patients who had the same PGI-S score at Baseline and Week 26 (ADSD *n* = 81; ANSD *n* = 70); ‘Worsened’ is defined as patients who reported at least a 1-point worsening on the PGI-S between Baseline and Week 26 (ADSD *n* = 15; ANSD *n* = 11); To ensure visibility, an offset of 0.1 is applied to change scores between 0.0 and 0.1 and an offset of -0.1 is applied to values lower than 0.0 but higher than -0.1. Within-group effect sizes were calculated as the mean change in ADSD and ANSD weekly summary score over time divided by the Baseline SD. Between-group effect sizes (defined as the difference in ADSD and ANSD weekly summary score between the two groups divided by the pooled SD at Baseline) were estimated between the improvement/worsening group and the ‘no change’ groups; effect sizes were assessed as small (d = 0.2), medium (d = 0.5), or large (d = 0.8), according to thresholds suggested by Cohen [17]. ADSD, Asthma Daytime Symptom Diary; ANSD, Asthma Nighttime Symptom Diary; PGI-S, Patient Global Impression of Severity; RCT, randomized controlled trial; SD, standard deviation

Most of the patients in the RCT sample who showed improvement (of at least 1 point) on the PGI-S by Week 26 also showed an improvement on their ADSD weekly summary score (i.e., lower score at Week 26 relative to Baseline, and hence lower symptom severity), whereas patients who showed no change on the PGI-S generally reported ADSD change weekly summary scores close to zero (Fig. 4). Differences in change between these PGI-S-defined groups were of a moderate-to-large effect size (d ≥ 0.68), and overall between-group comparisons were statistically significant at *p* < 0.001. Similar findings were observed with the RWS.

### WPMC

**Fig. 5:**
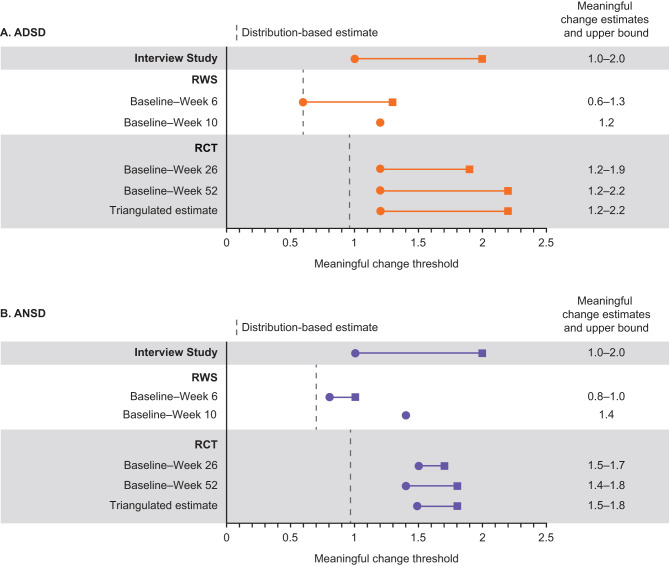
Estimated WPMC (clinically important response thresholds) for (**A**) ADSD/(**B**) ANSD. Circles show the meaningful change estimate with squares indicating the upper bound for each estimate; distribution-based estimates reflect the standard error of measurement as the lower limit; dashed lines indicate distribution-based estimates. ADSD, Asthma Daytime Symptom Diary; ANSD, Asthma Nighttime Symptom Diary, RCT, randomized controlled trial; RWS, real-world study; WPMC, within-patient meaningful change

For both the RWS and RCT, triangulation of anchor-based insight (supplemented with visualization and distribution-based approaches) was used in estimating candidate values for defining WPMC. In the RWS, this triangulation process supported inference of a WPMC threshold for within-patient improvement that ranged from 0.6 to 1.3 for ADSD and from 0.8 to 1.4 for ANSD across 6- and 10-week intervals, with these ranges representing plausible values estimated across the anchors (Fig. 5, Table E5; adequacy evaluation of anchors shown in Table E6).

For the RCT, the results demonstrated consistency in the WPMC estimate and its corresponding range across the two intervals of interest (change to Week 26 and to Week 52); the triangulated estimate for WPMC threshold was 1.2 (with an upper bound of 2.2) for ADSD and 1.5 (with an upper bound of 1.8) for ANSD (Fig. 5; Table E7).

## Discussion

This research established empirical evidence across quantitative sources regarding the measurement properties of the ADSD and ANSD, indicating their suitability for use as an endpoint in research within a moderate-to-severe asthma and severe asthma with type 2 inflammation population. The assessments of structural validity supported a single-dimension scoring solution for both the ADSD and ANSD, both measures demonstrated acceptable internal consistency and good test-retest reliability, and convergent, divergent, and known-groups validity evidence were generated. Importantly, the ADSD and ANSD were found to be sensitive to change in this population, and thresholds for inferring WPMC were established (1.2 for the ADSD and 1.5 for the ANSD). Taken as a whole, this portfolio of work fulfils the FDA request for additional evidence regarding the longitudinal measurement properties of these measures, demonstrating that they are fit for use in both interventional and real-world settings.

While the structural validity evidence supported the inference of unidimensionality at Baseline in the RWS, the RMSEA fit index was comparatively high while both CFI and SRMR indicated acceptable fit. This may indicate some residual misfit at Baseline. This study prioritized converging evidence across indices, consistent with recommendations to evaluate CFA fit using complementary measures (e.g., an incremental index such as CFI alongside a residual-based index such as SRMR), rather than relying on a single cutoff. In addition, RMSEA is known to over-reject (i.e., indicate poor fit) in parsimonious models with small degrees of freedom [16], as RMSEA can be inflated even when other indices and parameter estimates support the model. Importantly, the strong item loadings, improvement in RMSEA at later RWS timepoints and replication of broadly similar fit patterns in the RCT together support a parsimonious unidimensional scoring interpretation, while acknowledging the Baseline RMSEA as a limitation of the initial model-data correspondence.

Internal consistency reliability for ADSD and ANSD was high, with Cronbach’s α ≥ 0.94. While item redundancy can be a consideration where α is high, the ADSD and ANSD were intentionally designed to capture closely related manifestations of symptom severity [7, 8]. Consistent with this interpretation, α-if-item-deleted showed no material improvement when any single item was removed (max increase ≈ 0.01; max decrease ≈ 0.02), and McDonald’s ω provided additional support for the inference of good reliability overall. Collectively, these findings are against the inference of item redundancy in either the ADSD or ANSD.

WPMC in the ADSD and ANSD was investigated in both the RWS and RCT studies. The results were robust, with similar estimates for WPMC observed across both study types, despite the differences in samples, setting, and assessment intervals. While the RCT setting could reasonably be expected to be the most appropriate context for assessing WPMC, it is encouraging that the performance of the tools was replicated in the RWS setting. It should be noted that in the RWS there was a 3-week break before the final assessment. Despite this, the ADSD and ANSD demonstrated consistent psychometric measurement properties, suggesting that these tools can be used at intermittent intervals. This is important in the context of clinical trial design, whereby patient burden can be reduced by not administering every measure at all study visits; however, it should be balanced against the fact that this would result in less detailed longitudinal data due to the reduced data collection frequency and notable logistical challenges, such as increased risk of attrition due to temporary disengagement.

The findings from this study build on previous research demonstrating the content validity and cross-sectional measurement properties of the ADSD and ANSD, following initial tool development in a US population [7, 8]. That study generated strong cross-sectional support for the reliability and validity of the ADSD and ANSD items and scores derived from both measures. Here, we have replicated the cross-sectional evidence for the ADSD and ANSD in a RWS.

The 2019 FDA qualification of the ADSD and ANSD supported their use in drug development and regulatory review; however, it advised that before the qualification statement could be expanded to include the recommended use of the ADSD and ANSD as secondary or co-primary endpoints, additional evaluation on the longitudinal measurement properties (specifically, the ability to detect change) and the interpretation of WPMC in scores were required [9]. The psychometric evidence generated through the present evaluation has shown that the ADSD and ANSD are useful and additive, providing interpretable longitudinal data and an empirical basis for the interpretation of WPMC [9]. This study is part of a wider evidence generation exercise and builds on the evidence from a counterpart qualitative study that recently also demonstrated new support for the content validity of these instruments.

The longitudinal nature of this study is a strength which has been discussed above; another strength is the broad geographical population (drawn from the US, UK, and Germany). However, there are some limitations. The observational and noninterventional nature of the RWS introduces challenges for the assessment of WPMC and responsiveness, though this limitation is mitigated by concurrent evaluation of RCT data. The RWS was completed during the COVID-19 pandemic; patients with suspected or confirmed COVID-19 infection were excluded from psychometric analyses from the point at which the infection was first reported. While this minimized confounding due to comorbidity, it may also impact the generalizability of results. In the RCT, there was a drop in assessment completion over time. Additionally, the PGI-S was collected less frequently than the ADSD and ANSD (spanning intervals of 6 weeks or longer), posing some challenges in identifying stable patients for the evaluation of test-retest reliability in the RCT component of this work. Finally, generalizability should be interpreted in light of the differences between the two samples: the RWS comprised adults recruited online with moderate-to-severe asthma, whereas the RCT enrolled a clinically characterized patient population with severe asthma with type 2 inflammation (including a smaller adolescent subgroup). In addition, assessment of cross-cultural measurement equivalence was partially limited as small country-specific sample sizes (*n* < 50) for Germany required exclusion of this subset from the DIF analyses, limiting conclusions about measurement invariance in that setting.

These results provide evidence of validity of these measures in adult patients with moderate-to-severe asthma and severe asthma with type 2 inflammation. Future research in this area should seek to replicate these findings in differing patient populations with respect to demographics, ethnicity, culture, and disease profile, and to confirm that the thresholds for WPMC established in this study are performant in other contexts. Finally, exploratory work using scores from the ADSD and ANSD to inform other study outcomes such as symptom-free days would be beneficial.

## Conclusion

These studies, with different modalities of an RWS and an RCT, expand on the existing evidence of reliability and validity for the ADSD and ANSD PRO measures, previously qualified by the FDA for use in drug development studies, while also addressing a number of current evidence gaps [9]. The tools were found to be sensitive to change in this population, and thresholds for WPMC were established. Based on the values derived from the RCT analysis, the authors recommend thresholds for WPMC of 1.2 and 1.5 for the ADSD and ANSD, respectively.

As a measure of asthma symptom severity, the ADSD and ANSD are valuable additions to existing PRO assessments in asthma research. These data support the use of the ADSD and ANSD as standardized efficacy endpoint measures in confirmatory clinical studies and provide supportive information for real-world applicability to help inform on asthma symptoms and guide clinical management.

## Electronic Supplementary Material

Below is the link to the electronic supplementary material.

Supplementary Material 1

## Data Availability

GSK makes available anonymized individual patient data and associated documents from interventional clinical studies that evaluate medicines, upon approval of proposals submitted to: https://www.gsk-studyregister.com/en/.

## Abbreviations

ACQ: Asthma Control Questionnaire
ACT: Asthma Control Test
ADSD: Asthma Daytime Symptom Diary
ANCOVA: Analysis of covariance
ANSD: Asthma Nighttime Symptom Diary
CFA: Confirmatory factor analyses
CFI: Comparative fit index
DIF: Differential item functioning
EQ-5D-3L: EuroQoL 5-Dimensions 3-Levels
eCOA: Electronic clinical outcome assessment
FDA: US Food and Drug Administration
GINA: Global Initiative for Asthma
ICC: Intraclass correlation coefficient
PGI-C: Patient Global Impression of Change
PGI-S: Patient Global Impression of Severity
PRO: Patient-reported outcome
PROMIS SF: Patient-Reported Outcomes Measurement Information System Short Form
RCT: Randomized controlled trial
RMSEA: Root mean square error of approximation
RWS: Real-world study
SD: Standard deviation
SGRQ: St George’s Respiratory Questionnaire
SNOT-22: Sino-Nasal Outcome Test-22
SRMR: Standardized root mean residual
WPMC: Within-patient meaningful change
