# Supplementary material for: Validity, reliability, and longitudinal measurement properties of the Asthma Daytime and Nighttime Symptom Diaries in patients with moderate-to-severe asthma
Source: J Patient Rep Outcomes. 2026 May 20;10:89. doi: 10.1186/s41687-026-01066-5 (PMC13219701; doi:10.1186/s41687-026-01066-5)
Supplement: Supplementary file 1 — Supplementary Material 1 [file 41687_2026_1066_MOESM1_ESM.docx]

# Validity, reliability, and longitudinal measurement properties of the Asthma Daytime and Nighttime Symptom Diaries in patients with moderate-to-severe asthma

**Supplementary methods**

*Convergent and divergent validity*

For PGI-S, known groups definitions included ‘No symptoms’ (comprising “No symptoms” responses), ‘Medium severity’ (comprising “Mild” and “Moderate” responses) and ‘High severity’ (comprising “Severe” and “Very severe” responses). For ACT, they included ‘Poorly controlled’ (<20 total score) and ‘Well controlled’ (≥20 total score). For SGRQ, groupings were based on the items “How would you describe your chest condition”. PROMIS SF v1.0 Sleep Disturbance items (ANSD only) comparisons were completed cross-sectionally for groupings based on the following statements: “My sleep quality was…” and “I had problems with my sleep.” In the RCT, patient categories were defined using PGI-S scores and a selected SGRQ, plus a sleep-relevant SNOT-22 item score (ANSD only) at Baseline, Week 26, and Week 52. PGI-S categories were as described above for the RWS. SGRQ included item 3 “Shortness of breath” (5 categories), while SNOT-22 included item 15 “Lack of a good night’s sleep” (6 categories; ANSD only.)

**Supplementary results**

*Differential item functioning (DIF)*

Prior to multiple testing of the COVID-19 group comparison, an initial suggestion of uniform DIF was observed on two items on the ADSD: ‘Difficulty breathing’ (β=-1.01, p=0.009) and ‘Wheezing’ (β=1.01, p=0.021). Following adjustment of the false discovery rate to account for the number of tests simultaneously performed, neither DIF remained statistically significant and the corresponding items on the ANSD showed no indication of DIF. DIF (uniform or nonuniform) was not observed elsewhere for the COVID-19 group comparison.

For the GINA therapy level comparison, prior to multiple testing, an initial indication of uniform DIF was observed for the two ANSD items measuring chest symptoms: ‘Chest tightness’ (β=1.14, p=0.006) and ‘Chest pain’ (β=1.07, p=0.028), indicating that patients receiving step 4 therapy may have been more likely than those receiving step 5 therapy to report higher severity on these items for the same level of overall asthma severity. There was also an initial indication of uniform bias in the opposite direction (i.e., greater likelihood of higher scoring by patients with step 5 independent of overall asthma severity) on the ‘Cough’ item (β=-0.77, p=0.035). None of the ANSD estimates were deemed significant following adjustment of the false discovery rate to account for the number of tests simultaneously performed. Additionally, none of the corresponding items on the ADSD showed DIF.

No evidence of either uniform or nonuniform DIF was detected between the USA- and UK-based patients.

**Supplementary Tables and Figures**

*Table E1. Eligibility criteria*

| **Eligibility Criteria** | **RWS**  **(Study 214135)** | **RCT**  **(Study 217460)** |
| --- | --- | --- |
| Patient age and country | ≥16 years at the time of enrollment in the study  Germany, United Kingdom, and United States | ≥12 years at the time of enrollment*  Canada, China, Czechia, Germany*, Spain, France, United Kingdom, Ireland, Italy, Poland, Russia, United States |
| Diagnosis | Diagnosed with asthma by a physician at least  2 years prior (self-reported) | Diagnosis of asthma for ≥2 years that meets the National Heart, Lung, and Blood Institute guidelines (NHLBI, 2007) or GINA guidelines  (GINA, 2020) and evidence of type 2 inflammation (i.e. peripheral blood eosinophil count ≥300 cells/μL in the last 12 months or ≥150 cells/μL at screening) |
| Medication | Prescribed GINA 2020 step 3─5 medications at the time of enrollment defined as:  use in the preceding 3 months of a low-, or medium- or high-dose inhaled corticosteroid (ICS) and a long-acting beta agonist (LABA) ± an add-on controller therapy, with the latter add-on including an inhaled long-acting muscarinic antagonist (LAMA), and/or oral leukotriene receptor antagonist (LTRA), and/or oral corticosteroids (OCS), and/or a biologic therapy for asthma (anti-immunoglobin E; anti-interleukin(IL)-5/anti-interleukin-5 receptor or anti-IL4R alpha) | Well-documented requirement for regular treatment with medium- to high-dose ICS (in the 12 months before Visit 1 with or without maintenance OCS). The maintenance ICS dose must be ≥440 mcg fluticasone propionate hydrofluoroalkane (HFA) daily, or clinically comparable and receiving additional controller medication, besides ICS, for at least 3 months (e.g., LABA, LAMA, LTRA, or theophylline). Patients treated with medium-dose ICS also needed to be treated with LABA to qualify for inclusion |
| Disease history | Experienced at least one severe exacerbation in the previous year (for ≥50% of recruited patients) | Confirmed history of ≥2 exacerbations requiring treatment with systemic corticosteroids in the 12 months before Visit 1, despite the use of medium- to high-dose ICS, airflow obstruction (for patients >18 years, a pre-bronchodilator forced expiratory volume in 1 second [FEV_1_] <80% predicted; for patients 12–17 years, a pre-bronchodilator FEV_1_ <90% predicted or FEV_1_:forced vital capacity ratio <0.8 recorded at Visit 1) |
| Other | Owned or had access to a device that was suitable to download the study electronic clinical outcome assessment (eCOA) app |  |
| Exclusion criteria | Had a disease/condition, other than asthma, that had a significant impact on the patient’s experience with respiratory symptoms, including current infection (either COVID-19 or other infection); bronchiectasis; pulmonary fibrosis; bronchopulmonary aspergillosis; or diagnoses of emphysema or chronic bronchitis (chronic obstructive pulmonary disease other than asthma) or a history of lung cancer.  Current and/or former^†^ smokers of ≥10 pack years were excluded | Pre-existing clinically significant cardiac, endocrine, autoimmune, metabolic, neurological, renal, gastrointestinal, hepatic, hematologic, or any other system abnormalities that are uncontrolled with standard treatment  Current and/or former smokers of ≥10 pack years were excluded  Patients who have received mepolizumab, reslizumab, or benralizumab within 12 months before Visit 1 or who have a previous documented  failure with anti-IL-5/5R therapy7  Patients who have received omalizumab or dupilumab within 130 days before Visit 1  Patients who have received any monoclonal antibody within 5 half-lives of Visit 1. Authorized treatments for COVID-19 are permitted |

*For countries where local regulations or the regulatory status of study medication permit enrollment of adults only, patients recruited were ≥18 years of age; ^†^a former smoker was defined as a patient who has stopped smoking for at least the past 6 months.

*Table E2. Overview of PROs used in the RWS and RCT*

| **PRO** | **Assessed in:** | **Measure details** |
| --- | --- | --- |
| **ACQ-5**  (Asthma Control Questionnaire 5-item) | RCT | The ACQ-5 is a self-administered 5-item PRO with a 1-week recall period designed to provide a quick and easy measure of a patient’s asthma control [1]. Response items are equally weighted and range from 0 (excellent asthma control) to 6 (extremely poor asthma control). A total score is calculated, with higher scores indicating well-controlled asthma [1]. |
| **ACT**  (Asthma Control Test) | RWS | The ACT is a 5-item, disease-specific PRO assessing the frequency of shortness of breath and general asthma symptoms, rescue medication use, effects of asthma on daily functioning, and overall self-assessment of asthma control. It is designed to identify patients with poorly controlled asthma and is intended to be used in patients aged more than 12 years [2].  Higher ACT scores show greater asthma control, with scores above 20 indicating well-controlled asthma [3]. |
| **EQ-5D-3L**  (EuroQoL 5-Dimensions  3-Levels) | RWS | The EQ-5D-3L is a 6-item health status measure assessing overall health and 5 specific domains (mobility, self-care, usual activities, pain/discomfort, and anxiety/depression). Domains are rated according to three standardized categorizations, and the final question is a Visual Analog Scale ranking health status from ‘Best health imaginable’ to ‘Worst health imaginable’ [4]. |
| **PGI-C**  (Patient Global Impression of Change) | RWS (PGI-C Day/PGI-C Night) and RCT | The PGI-C is a single-item measure, developed by the Critical Path Institute, capturing a patient’s perception of change in the severity of their asthma symptoms since the start of a study rated using a 5-point ordinal scale. For the RWS, two forms of the PGI-C were used, one collected in the evening to assess perception of change in daytime symptoms (PGI-C Day), and the other collected in the morning to assess perception of change in nighttime symptoms (PGI-C Night) [5]. |
| **PGI-S**  (Patient Global Impression of Severity) | RWS and RCT | The PGI-S is a single-item measure, developed by the Critical Path Institute, capturing a patient’s perception of asthma severity over the last 7 days rated using a 5-point ordinal scale. For the RWS, two forms of the PGI-S were used, one administered in the evening to assess earlier daytime symptom severity (PGI-S Day) and the other collected in the morning to assess prior nighttime symptom severity (PGI-S Night) [5]. |
| **PROMIS SF Sleep Disturbance 8a**  (Patient-Reported Outcomes Measurement Information System Short Form Sleep Disturbance 8a) | RWS | PROMIS SF Sleep Disturbance 8a is a generic (as opposed to disease-specific) 8-item questionnaire assessing self-reported sleep quality, sleep depth, and restoration associated with sleep [6,7] and was validated in healthy adults and adolescents (aged 10 to 18 years) [6,8]. It has a 7-day recall period, and items are assessed using a 5-point scale to rate the severity of sleep disturbance. Higher scores indicate a greater severity of sleep disturbance [6]. |
| **PROMIS Fatigue items**  (Patient-Reported Outcomes Measurement Information System Fatigue item) | RCT | The PROMIS Fatigue item bank includes multiple items to assess fatigue, with each item rated on a 5-point categorial response scale [9,10]. In the present study, items were used in the psychometric evaluation as individual measures, rather than as a summated/aggregated scale. |
| **SGRQ**  (St George’s Respiratory Questionnaire) | RWS and RCT | The SGRQ is a 50-item PRO measure composed of two parts designed to assess the impact of respiratory difficulties on overall health and daily life in patients with asthma and chronic obstructive pulmonary disease [11,12]. Part 1 contains several scales and assesses the frequency and severity of symptoms with a current and 4-week recall period. Part 2 assesses activities that cause or are limited by breathlessness and also contains the impact components (social functioning, psychological disturbances resulting from airways disease). It refers to the current state without a defined recall period and includes both dichotomous response options (true/false) and one 4-point Likert scale [11,12].  Items within the measure are given a statistical weight providing an estimate of the distress associated with the symptom. Total scores are calculated to summarize the impact of the disease on overall health status [11,12]. |
| **SNOT-22**  (Sino-Nasal Outcome Test-22) | RCT | SNOT-22 is a 22-item PRO measure for the assessment of disease-specific quality of life of chronic rhinosinusitis (with or without nasal polyposis). It contains questions about a broad range of health and health-related quality of life aspects, each of which is rated on a 6-point categorical response scale according to the severity of the problem, limitation, or consequence the patient has experienced over the previous 2 weeks [13]. |

*Table E3. Confirmatory factor analysis for proposed ADSD and ANSD factorial models–factor loadings in RWS/RCT*

RWS

|  | Item Loadings | | | | | |
| --- | --- | --- | --- | --- | --- | --- |
|  | ADSD | | | ANSD | | |
| Asthma Symptom | Baseline | Week 6 | Week 10 | Baseline | Week 6 | Week 10 |
| Breathing | 0.93 | 0.94 | 0.94 | 0.93 | 0.95 | 0.94 |
| Wheezing | 0.86 | 0.89 | 0.92 | 0.88 | 0.91 | 0.87 |
| Shortness of breath | 0.91 | 0.95 | 0.95 | 0.93 | 0.93 | 0.95 |
| Chest tightness | 0.89 | 0.91 | 0.94 | 0.91 | 0.92 | 0.93 |
| Chest pain | 0.79 | 0.83 | 0.83 | 0.81 | 0.74 | 0.87 |
| Cough | 0.68 | 0.69 | 0.76 | 0.61 | 0.77 | 0.73 |

RCT

|  | Item Loadings | | | | | |
| --- | --- | --- | --- | --- | --- | --- |
|  | ADSD | | | ANSD | | |
| Asthma Symptom | Baseline | Week 26 | Week 52 | Baseline | Week 26 | Week 52 |
| Breathing | 0.96 | 0.97 | 0.97 | 0.95 | 0.97 | 0.98 |
| Wheezing | 0.93 | 0.91 | 0.88 | 0.92 | 0.89 | 0.85 |
| Shortness of breath | 0.97 | 0.98 | 0.97 | 0.96 | 0.98 | 0.98 |
| Chest tightness | 0.92 | 0.93 | 0.92 | 0.92 | 0.90 | 0.87 |
| Chest pain | 0.84 | 0.88 | 0.86 | 0.85 | 0.83 | 0.81 |
| Cough | 0.86 | 0.84 | 0.84 | 0.85 | 0.83 | 0.83 |

Goodness-of-fit indices for one-factor CFA models approached or exceeded recommended thresholds (CFI ≥0.95; RMSEA ≤0.06; SRMR ≤0.08) for the ADSD and ANSD.

ADSD, Asthma Daytime Symptom Diary; ANSD, Asthma Nighttime Symptom Diary; CFA, confirmatory factor analysis; CFI, comparative fit index; RCT, randomized controlled trial; RMSEA, root mean square error of approximation; RWS, real-world study; SRMR, standardized root mean residual.

*Table E4. Additional internal consistency results for the ADSD and ANSD total scores in the RWS and RCT*

*RWS*

| Timepoint | | | ADSD total score | ANSD total score |
| --- | --- | --- | --- | --- |
| Baseline | N | | 205 | 191 |
|  | Cronbach's α if variable deleted | Item 1: Difficulty breathing | 0.92 | 0.94 |
|  |  | Item 2: Wheezing | 0.93 | 0.95 |
|  |  | Item 3: Shortness of breath | 0.93 | 0.94 |
|  |  | Item 4: Chest tightness | 0.93 | 0.94 |
|  |  | Item 5: Chest pain | 0.94 | 0.95 |
|  |  | Item 6: Cough | 0.95 | 0.96 |
| Week 6 | N | | 174 | 162 |
|  | Cronbach's α if variable deleted | Item 1: Difficulty breathing | 0.94 | 0.95 |
|  |  | Item 2: Wheezing | 0.95 | 0.95 |
|  |  | Item 3: Shortness of breath | 0.94 | 0.95 |
|  |  | Item 4: Chest tightness | 0.94 | 0.95 |
|  |  | Item 5: Chest pain | 0.95 | 0.96 |
|  |  | Item 6: Cough | 0.96 | 0.97 |
| Week 10 | N | | 163 | 163 |
|  | Cronbach's α if variable deleted | Item 1: Difficulty breathing | 0.95 | 0.96 |
|  |  | Item 2: Wheezing | 0.96 | 0.96 |
|  |  | Item 3: Shortness of breath | 0.95 | 0.96 |
|  |  | Item 4: Chest tightness | 0.96 | 0.96 |
|  |  | Item 5: Chest pain | 0.96 | 0.96 |
|  |  | Item 6: Cough | 0.97 | 0.97 |

RCT

| Baseline | N | | 328 | 300 |
| --- | --- | --- | --- | --- |
|  | Cronbach's α if variable deleted | Item 1: Difficulty breathing | 0.942 | 0.942 |
|  |  | Item 2: Wheezing | 0.943 | 0.944 |
|  |  | Item 3: Shortness of breath | 0.939 | 0.941 |
|  |  | Item 4: Chest tightness | 0.944 | 0.942 |
|  |  | Item 5: Chest pain | 0.953 | 0.953 |
|  |  | Item 6: Cough | 0.952 | 0.952 |
| Week 26 | N | | 189 | 178 |
|  | Cronbach's α if variable deleted | Item 1: Difficulty breathing | 0.952 | 0.939 |
|  |  | Item 2: Wheezing | 0.949 | 0.938 |
|  |  | Item 3: Shortness of breath | 0.948 | 0.936 |
|  |  | Item 4: Chest tightness | 0.950 | 0.939 |
|  |  | Item 5: Chest pain | 0.954 | 0.947 |
|  |  | Item 6: Cough | 0.962 | 0.953 |
| Week 52 | N | | 101 | 77 |
|  | Cronbach's α if variable deleted | Item 1: Difficulty breathing | 0.944 | 0.929 |
|  |  | Item 2: Wheezing | 0.952 | 0.937 |
|  |  | Item 3: Shortness of breath | 0.948 | 0.929 |
|  |  | Item 4: Chest tightness | 0.947 | 0.936 |
|  |  | Item 5: Chest pain | 0.952 | 0.950 |
|  |  | Item 6: Cough | 0.957 | 0.946 |

ADSD, Asthma Daytime Symptom Diary; ANSD, Asthma Nighttime Symptom Diary; N, Number of patients; RCT, randomized controlled trial; RWS, real-world study.

*Table E5. Candidate thresholds for evaluating within-patient meaningful improvement (clinically important responders) in the RWS and RCT*

*RWS*

| **Scale** | **Time interval** | **Anchor** | **Anchor version** | **Anchor group** | **Group mean change** | **ROC (‘improved vs other’)** | **SEM** |
| --- | --- | --- | --- | --- | --- | --- | --- |
| ADSD | Up to 6 weeks | PGI-S | 7-level | Improved one category | -0.93 | -0.64 | 0.62 |
|  |  | PGI-C | 5-level | Improved two categories | -1.26 | Not included as AUC was too low |  |
|  | Up to 10 weeks | PGI-S | 7-level | Improved one category | -1.18 | -0.4 |  |
| ANSD | Up to 6 weeks | PGI-S | 7-level | Improved one category | -0.81 | Not included as AUC was too low | 0.69 |
|  |  | PGI-C | 5-level | Improved two categories | -1.04 | Not included as AUC was too low |  |
|  | Up to 10 weeks | PGI-S | 7-level | Improved one category | -0.96 | -0.17 |  |
|  |  | PGI-C | 5-level | Improved one category | -0.49 | Not included as AUC was too low |  |
|  |  |  |  | Improved two categories | -1.36 |  |  |

RCT

| **Scale** | **Time interval** | **Anchor** | **Anchor version** | **Anchor group** | **Group mean change** | **ROC (‘improved vs other’)** | **SEM** |
| --- | --- | --- | --- | --- | --- | --- | --- |
| ADSD | Up to 26 weeks | PGI-S | 7-level | Improved one category | -1.20 | - | 0.410 |
|  |  |  | 3-level | Improved | -1.90 | - |  |
|  |  | PGI-C | 3-level | Improved | -1.51 | - |  |
|  | Up to 52 weeks | PGI-S | 7-level | Improved one category | -1.20 | - |  |
|  |  |  | 3-level | Improved | -1.90 |  |  |
|  |  | PGI-C | 5-level | Improved two categories | -2.2 | - |  |
| ANSD | Up to 26 weeks | PGI-S | 7-level | Improved one category | -1.50 | - | 0.375 |
|  |  |  | 3-level | Improved | -1.70 | - |  |
|  |  | PGI-C | - | - | Correlation with anchor inadequate | - |  |
|  | Up to 52 weeks | PGI-S | 7-level | Improved one category | -1.40 | - |  |
|  |  | PGI-C | 3-level | Improved | -1.80 | - |  |
|  |  | PGI-C | - |  | *See footnote | - |  |

* None of PGI-C defined improvement groups (with either the collapsed or uncollapsed version of the anchor) showed adequate differentiation from the PGI-C defined no change group.

Group mean change values and ROC change thresholds that do not exceed the distribution-based

estimate (i.e., the SEM) are shaded in gray as these do not meet the requirements for use in evaluating the meaningfulness of within-patient improvement as measured by the ADSD/ANSD. AUC, area under curve; PGI-C, Patient Global Impression of Change; PGI-S Patient Global Impression of Severity; ROC, receiver operating characteristic; SEM, standard error of measurement.

*Table E6. PGI-S and PGI-C anchors adequacy evaluation for assessing within-patient meaningful change from Baseline to Week 26 and Week 52 in ADSD and ANSD weekly summary scores in RCT*

| **Total Score** | **Time interval** | **Anchor** | **Anchor version** | **Correlation Coefficient** |
| --- | --- | --- | --- | --- |
| ADSD | CFB to  Week 26 | PGI-S | Uncollapsed 9-categories | 0.516 |
|  |  |  | Collapsed 7-categories | 0.536 |
|  |  |  | Collapsed 3-categories | 0.503 |
|  |  | PGI-C | Uncollapsed 5-categories | 0.295 |
|  |  |  | Collapsed 3-categories | 0.315 |
|  | CFB to  Week 52 | PGI-S | Uncollapsed 9-categories | 0.563 |
|  |  |  | Collapsed 7-categories | 0.612 |
|  |  |  | Collapsed 3-categories | 0.587 |
|  |  | PGI-C | Uncollapsed 5-categories | 0.429 |
|  |  |  | Collapsed 3-categories | 0.386 |
| ANSD | CFB to Week 26 | PGI-S | Uncollapsed 9-categories | 0.540 |
|  |  |  | Collapsed 7-categories | 0.569 |
|  |  |  | Collapsed 3-categories | 0.536 |
|  |  | PGI-C | Uncollapsed 5-categories | 0.239 |
|  |  |  | Collapsed 3-categories | 0.249 |
|  | CFB to  Week 52 | PGI-S | Uncollapsed 9-categories | 0.503 |
|  |  |  | Collapsed 7-categories | 0.568 |
|  |  |  | Collapsed 3-categories | 0.553 |
|  |  | PGI-C | Uncollapsed 5-categories | 0.412 |
|  |  |  | Collapsed 3-categories | 0.302 |

*Correlation coefficients are Pearson or Spearman correlations for the uncollapsed groups of PGI-S and polychoric correlations for collapsed groups of PGI-S and collapsed and uncollapsed groups of PGI-C. The change scores from Baseline are based on the difference between weekly summary scores of all nonmissing scores at Week 26 and 52 minus the weekly summary scores at Baseline.*

*CFB, Change from Baseline; PGI-C, Patient Global Impression of Change; PGI-S, Patient Global Impression of Severity.*

*Table E7. Summary of clinically important response threshold proposals for evaluating WPMC amongst a patient population with moderate-to-severe asthma*

| **Instrument** | **Baseline to Week 26 evaluation** | **Baseline to Week 52 evaluation** | **Overall recommendation** |
| --- | --- | --- | --- |
| ADSD | 1.2, with an upper bound of 1.9 | 1.2, with an upper bound of 2.2 | 1.2, with an upper bound of 2.2 |
| ANSD | 1.5, with an upper bound of 1.7 | 1.4, with an upper bound of 1.8 | 1.5, with an upper bound of 1.8 |

ADSD, Asthma Daytime Symptom Diary; ANSD, Asthma Nighttime Symptom Diary; WPMC, within-patient meaningful change.

**Supplementary References**

1. Juniper EF, Svensson K, Mörk AC, Ståhl E (2005). Respir Med 99 (5):553-558. doi:10.1016/j.rmed.2004.10.008

2. Nathan RA, Sorkness CA, Kosinski M, Schatz M, Li JT, Marcus P, Murray JJ, Pendergraft TB (2004). J Allergy Clin Immunol 113 (1):59-65. doi:10.1016/j.jaci.2003.09.008

3. Schatz M, Mosen DM, Kosinski M, Vollmer WM, Magid DJ, O'Connor E, Zeiger RS (2007). Am J Manag Care 13 (12):661-667

4. EuroQol Research Foundation (2018) EQ-5D-3L User Guide. EuroQol Research Foundation https://euroqol.org/wp-content/uploads/2021/01/EQ-5D-3LUserguide-14-0421.pdf. Accessed March 2024

5. Eremenco S, Chen WH, Blum SI, Bush EN, Bushnell DM, DeBusk K, Gater A, Nelsen L, Coons SJ, Subcommittee PROCsC (2022). Qual Life Res 31 (12):3501-3512. doi:10.1007/s11136-022-03180-5

6. Buysse DJ, Yu L, Moul DE, Germain A, Stover A, Dodds NE, Johnston KL, Shablesky-Cade MA, Pilkonis PA (2010). Sleep 33 (6):781-792. doi:10.1093/sleep/33.6.781

7. Yu L, Buysse DJ, Germain A, Moul DE, Stover A, Dodds NE, Johnston KL, Pilkonis PA (2011). Behav Sleep Med 10 (1):6-24. doi:10.1080/15402002.2012.636266

8. Hanish AE, Lin-Dyken DC, Han JC (2017). Nurs Res 66 (3):246-251. doi:10.1097/NNR.0000000000000217

9. Christodoulou C, Junghaenel DU, DeWalt DA, Rothrock N, Stone AA (2008). Qual Life Res 17 (10):1239-1246. doi:10.1007/s11136-008-9402-x

10. Cella D, Lai JS, Jensen SE, Christodoulou C, Junghaenel DU, Reeve BB, Stone AA (2016). J Clin Epidemiol 73:128-134. doi:10.1016/j.jclinepi.2015.08.037

11. Jones PW, Forde Y (2009) St George’s Respiratory Questionnaire Manual. St George’s University London,

12. Paap MC, Brouwer D, Glas CA, Monninkhof EM, Forstreuter B, Pieterse ME, van der Palen J (2015). Qual Life Res 24 (1):67-79. doi:10.1007/s11136-013-0570-y

13. Hopkins C, Gillett S, Slack R, Lund VJ, Browne JP (2009). Clin Otolaryngol 34 (5):447-454. doi:10.1111/j.1749-4486.2009.01995.x
